# Engineered scPDL1-DM1 drug conjugate with improved *in vitro* analysis to target PD-L1 positive cancer cells and intracellular trafficking studies in cancer therapy

**DOI:** 10.1590/1678-4685-GMB-2018-0391

**Published:** 2020-01-17

**Authors:** Muhammad Kalim, Shenghao Wang, Keying Liang, Muhammad Saleem Iqbal Khan, Jinbiao Zhan

**Affiliations:** 1 Department of Biochemistry, Cancer Institute of the Second Affiliated Hospital, Zhejiang University, School of Medicine, Hangzhou, China.; 2 The China-US (Henan) Hormel Cancer Institute, Zhengzhou, Henan, China.

**Keywords:** Antibody-drug conjugate, biotherapeutics, cancer therapy, intracellular trafficking, PD-L1

## Abstract

Antibody-drug conjugates (ADC), precisely deliver a cytotoxic agent to antigen-expressing tumor cells by using specific binding strategies of antibodies. The ADC has shown the ability of potent bio-therapeutics development but indefinite stoichiometric linkage and full-length antibody penetration compromised the field of its advancement. Single chain variable fragments convention instead of the full-length antibody may overcome the challenge of rapid penetration and internalization. Programmed cell death ligand-1 interaction with PD-1 has recently revolutionized the field of immunotherapy. We systematically designed scPDL1-DM1 drug conjugate by linking scFv-PD-L1 proteins (scFv) with maytansinoids (DM1) cytotoxic agent through succinimidyl trans-4-maleimidylmethyl cyclohexane-1- carboxylate (SMCC) linker. Binding affinity was confirmed by immunocytochemistry, spectrophotometry and gel electrophoresis analysis. The scPDL1-DM1 showed specific binding with PD-L1 positive tumor cells and retained *in vitro* anti-cell proliferation activity. The intracellular trafficking of the drug was evaluated in A549 cancer cell lines, and maximum trafficking was observed after two hours of incubation. The generated drug can be utilized as a potent tool for site-specific conjugation, predicting specificity *in vitro* activities with extended range against PD-L1 positive cancer cells and can be utilized for further *in vivo* testing and clinical therapeutics development.

## Introduction

Targeted chemotherapy surges discerning the delivery of toxic drugs to tumor cells by conjugating them with monoclonal antibodies (mAbs). Antibody-drug conjugates combine the ultimate possessions of both antibodies and drugs to target precisely the tumor cells. Antibody mono-therapy often lacks elevated therapeutic index and limits immune induction, alternatively, antibodies can be joined with cytotoxic drugs to efficiently reduce systematic toxicity and enhance targeted delivery to tumor cells (Dal Corso *et al.*, 2018). Various cytotoxic conjugated agents are now in clinical use as calicheamicin and duocarmycin (DNA alkylation agents), auristatin and maytansinoids (microtubules disruption mediators), and DNA binding anthracyclines (Beck and Reichert, 2014).

Generally, lysine and cysteine sulfhydryl chains help in the conjugation of drugs to antibodies by reducing disulfide bonds in the development of antibody-drug conjugates (ADC). This heterogeneity facilitates binding of 2, 4 and 8 drug residues to targeted antibody and was reported in vivo potency in mice model. The antibody loaded with the maximum number of drugs might be rapidly clear showing low potency and instability or disruption. The consistency of ADC and its counterpart is laborious work to control potency in vivo studies. Four different ADC molecules were approved by the US Food and Drug Administration and more than 100 ADC molecules are in clinical evaluation to treat the solid tumor (Lambert and Morris, 2017; Yu *et al.*, 2015). Successful optimization of ADC depends on the selection of antibodies, the stability of linkers and potency of toxic drugs consequently to enhance tumor suppression. Additionally, the developmental progress of ADC depends extensively on better knowledge of antibody components of ADC and its trafficking in the intracellular region of targeted tumor cells (Dal Corso *et al.*, 2018). Several studies indicate that engineered antibody can be utilized to exploit the endosomal pathways that provide a substantial clove for future studies and better designing of ADC (Kalim *et al.*, 2017).

The experimental analysis provides knowledge of the intracellular process in greater aspects dissolves recent divergences and enhances the ability to select novel and efficient targets for ADCs attachment and enhance the existing ADC engineering strategies. Additional dynamic research analysis must be needed to parallel analysis, like studies of tumor cells toxicity, target receptors modification studies, cascade signaling analysis of receptors modulation by antibodies, and conjugates redesigning approaches.

Generations of scFv with highly desirable specificity and binding affinities are mostly reported from phage display technology (Pande *et al.*, 2010; Li and Caberoy, 2010). It is being considered a potent biological entity for the screening of targeted ligands that bind to the tumor surfaces, initially introduced in 1985 by Smith (Jemal *et al.*, 2010; Smith, 1985). The simplicity of scFvs can eradicate hurdles associated with whole antibodies in ADC conjugates, with a demonstration of superior penetration ability in tumor cells and tissues (Chames *et al.*, 2009; Beckman *et al.*, 2007; Yokota *et al.*, 1992). The reduced complexity of scFvs also allowed prokaryotic cells to produce desirable products efficiently in a short time and less maintenance of prokaryotic cells’ growth condition compared to antibody production by eukaryotic host machinery (Yokota *et al.*, 1992; Makrides, 1996). Recently, cancer targeted therapy has become one of the prominent hotlines in tumor eradication therapy. Highly specific carrier molecules are in practice to specifically deliver anti-tumor loaded drugs to target sites (Velasco-Velazquez *et al.*, 2014). Small peptide molecules may act as the most vital source of the carrier due to high specificity, structure simplicity, easy manipulation, and solid penetration ability to tumor cells (Bastien *et al.*, 2015).

We systematically designed scPDL1-DM1 drug conjugate by binding single-chain variable fragment (scFv) against programmed cell death ligands- 1 (PD-L1) with maytansinoids (DM1) cytotoxic agent through succinimidyl trans-4-maleimidylmethyl cyclohexane-1- carboxylate (SMCC) to develop scPDL1-DM1. Maytansinoids (DM1/DM4) inhibit tubulin polymerization and reported in various ADC conjugates for clinical/preclinical development that results in cell cycle arrest in G2/M phase through tubulin assembly inhibition and ultimately cell death (Dal Corso *et al.*, 2018, Weber *et al.*, 2015). The same SMCC-DM1 conjugation strategies were utilized by ado-trastuzumab emtansine (T-DM1, Kadcyla), an anti-HER2/3 drug conjugate that was approved by the US FDA for metastatic breast cancers treatment in 2013 (Lambert and Chari, 2014). Woitok *et al.* (2016) reported the fusion protein development by using two scFv molecules from humans and mice that recognized EGFR on HEK293T solid tumors. The fusion proteins were conjugated with auristatin F that specifically binds with targeted cells and triggers tumor cell cycle arrest (García-Alonso *et al.*, 2018). A similar study of regeneration of anti-CD22- scFv to humanized IgG1 antibody format was reported to design ADC in hematological malignancies (Sussman *et al.*, 2018).

In our study, we have reported the generation of scFv-PD-L1 drug conjugate (scPDL1-DM1) and in vitro activity analysis against different cancer cell lines. We also optimized the intracellular trafficking studies against A549 hepatocellular cancer cell lines using the newly developed drug. This conjugate has achieved its initial potency and needs efficient improvement to enhance direct tumor suppression and bio-therapeutics strategies enrichment.

## Material and Methods

### Reagents and cell lines

Tumor cell lines BEL-7402 (Hepatic Carcinoma), A549 (Adenocarcinoma, Lung Cancer), IK (Ishikawa, Endometrium cancer), LOVO (Colorectal cancer), and MDA453 cell lines were obtained from ATCC and were maintained in RPMI-1640 supplemented with 10% Fetal Bovine Serum. Cells were cultured in a humidified incubator with 5% CO_2_ at 37°C. The high-affinity scFv-PD-L1 single-chain proteins were previously engineered by our group using phage display technology and positive clones were selected by bio-panning strategies and successfully expressed in bacterial host cell machinery (Kalim *et al.*, 2019). Anti-PD-L1 IgG antibody and anti-6xHis Tag rabbit antibody (Cat No AB 10002) were from Life Science Production & Services, China. Rabbit anti-human IgG (H+L)-HRP (Cat No 6140-05; Lot No D2311-ZD51E) were from Southern Biotech USA) and goat anti-rabbit IgG-HRP (Cat No HA1001; Lot No G161011) were provided by Hangzhou HunAn Biotech Comp. China. All reagents, solutions, and buffers were maintained under high-grade purity and strict sterile condition.

### Generation of drug conjugate

SMCC (20 mM, 6.69 mg/mL) with final concentration of 1 mg/150 μl and DM1 (10 mM, 7.37 mg/mL) with final concentration of 1 mg/135.69 μl were dissolved in reaction buffer (DMSO). 20 fold volume of SMCC (12.12 μl) was conjugated with 10 fold of DM1 (36.36 μl) to make a total volume of 48.48 μl mixture. The final mixture was incubated at 20°C for 4 hours. The scFv-PD-L1 (1 mg/mL) proteins were adjusted with reaction buffer up to 6.7% and slowly mixed with the SMCC-DM1 mixture. The final fusion was slowly mixed at 20°C for 20 hours. The di-filtered fusion products were loaded to the Sephadex G-25 column to filter out the drug conjugate. Before filtration, the column was prepared by loading Sephadex G-25 beads slurry to 50 mL column and left for a night stay to acquire a specific settled position for beads. The column was constantly washed with 1X PBS (pH 7.2). The solution was loaded into the G-25 column and was filtered out with a flow rate of 0.5 mL/min. The filtrates were collected in 2 mL centrifuge tubes and conjugation reaction was monitored by Shimadzu-UV-2600 Spectrophotometer.

### Spectrophotometry analysis

The absorbance rates of filtered drug conjugates were conducted by Shimadzu-2600 spectrophotometer with a measurement range of 250 nm and 280 nm. The analysis was conducted with an accuracy of ± 0.05 nm wavelength repeatability by adopting low stray light diffraction grating. The SMCC-DM1-scFv PD-L1 mixture was filtered and loaded into the column. The conjugated ADC filtrate was eluted with 1X PBS (pH 7.2) at 0.5 mL/min flow rate in separate centrifuge tubes of 2 mL volume in total and absorbance was calculated against blank 1X PBS (pH 7.2) tube by spectrophotometric analysis.

### SDS-PAGE

The molecular weight of SMCC-DM1 was found 1072.618 g/moL with an approximate weight of 1 kDa. The purified scFv-PD-L1 protein molecular weight was found approximately 34 kDa. The drug conjugate was loaded on 12% SDS-PAGE to make sure the slight increase of 1 kDa on gel slice.

### In vitro activity determination

*In vitro* activity of the scPDL1-DM1 was assessed in BEL-7402 (Hepatic Carcinoma), A549 (Adenocarcinoma, Lung Cancer), IK (Ishikawa, Endometrium cancer), LOVO (Colorectal cancer) and MDA453 cancer cell lines in 96 wells plate at a ratio of 5000 cells per well with the different time frame. Cell viability analysis was calculated by using MTT assay. The assay assesses cell metabolic activity to measure the cytotoxicity of the drug. The cells were exposed to scPDL1-DM1 at different concentrations ranges from 1 - 0.0001 μg/mL and incubated with various concentrations of drug conjugate for 48, 60 and 72 hours. The reaction was stopped interval followed by the addition of 50 μl MTT reagent. The plates were gently shaken and incubated up to 4 hours. The supernatant was slowly removed and propanol was added to solubilize the formazan crystals. The absorbance was measured and growth inhibition was calculated using the formula:

% cell survival = {(Ta-Tb)/(Tc-Tb)} x 100 whereas Ta was tested absorbance, Tb was blank absorbance and Tc was control absorbance.

In each of two or three independent experiments, triplicate wells were prepared for each condition at each time point analysis and cell viability was calculated.

### Immunofluorescence

Tumor cell lines BEL-7402 (Hepatic Carcinoma), A549 (Adenocarcinoma, Lung Cancer), IK (Ishikawa, Endometrium cancer), LOVO (Colorectal cancer), and MDA453 cell lines were seeded onto glass coverslips at 2x10^4^/ml in regular serum-containing RPMI 1640 medium and incubated at 37°C until 80% confluence. Cells were washed gently with 1X PBS (pH 7.2) and fixed with 4% paraformaldehyde for 20 minutes. After washing three times, the drug conjugate was applied and incubated for one night at 4°C. Coverslips were further washed 3 times with ice-cold 1X PBS (pH 7.2) and loaded with FITC labeled rabbit anti-human IgG (H+L) antibody (dilution 1:1000) for 1 hour at 37°C. DAPI (dilution 1:1000) was added after three times washing (5 minutes in each wash) for 10 minutes and coverslips were mounted in an inverted position on the slide and were visualized under 63x oil immersion objective using Zeiss fluorescence microscope (Zeiss, Germany).

### Intracellular trafficking

A549 cells were inoculated on ice with drug conjugate for 60 minutes in 1mL of total volume in 1X PBS supplemented with 1% FBS. After washing, cells were incubated at 37°C for a different interval of times (0 min, 10 min, 30 min, 1 hr, 2 hrs, and 3 hrs). After incubation, cells were fixed and conjugated with FITC labeled rabbit anti-human IgG (H + L) antibody for 1 hour and analyzed for trafficking studies. Additional studies of fluorescence were also conducted to visualize the surface bindings by using scPDL1-DM1 and mouse monoclonal Lamp I antibody. The lamp I antibody was used for lysosome trafficking followed by incubation interval with FITC labeled rabbit anti-human IgG (H + L) and rabbit anti-mouse Alexa 488 antibody. Slides were loaded with DAPI for ten minutes and washed before mounting on slides. The slides were visualized under a 63x oil immersion objective using a Zeiss fluorescence microscope (Zeiss, Germany).

### Statistical Analysis

One way ANOVA analysis of variance by using GraphPad Prism 5 (GraphPad Software; Inc: La Jolla, CA) software was used to evaluate the cytotoxic activity of the drug. Values were expressed as the mean ± SEM and analyzed with Student’s t-test. The test samples were applied in triplicate. The significance level was set at p ≤ 0.05. All the data are presented as means ± SD. Image J and Excel spreadsheet were also used for graphical presentation.

## Results

### Development of scFv-PD-L1 drug conjugate

Antibody-drug conjugates were designed with maytansinoids (DM1) cytotoxic agent through succinimidyl trans-4-maleimidylmethyl cyclohexane-1- carboxylate (SMCC) thioether linker for active release of drug by intracellular reduction. The efficient site-specific conjugations of scFv-PD-L1 protein with SMCC-DM1 were conducted with the specification of light and heavy chains constant regions. Clinically ADC utilizes either auristatin or maytansine derivatives developed by Seattle Genetics and ImmunoGen. Reaction mixture stocks were created by dissolving separately 20 mM SMCC (0.448 mL) linker and 10 mM DM1 drug (0.502 mL) in DMSO. The processes were conducted under a sterilized fume hood and were mixed together along with scFv-PD-L1 proteins. The SMCC-DM1 mix was added to 1mg/mL protein concentrations and incubated further for 20 hours at 20°C to allow the binding of linker-drug attachment. Applying this approach allowed the suitability of variable and light chains of anti-PD-L1 to targeted site conjugates. The final conjugates were filtered before applying to preparative size exclusion chromatography on G-25 Sephadex column. The flow rate was kept constant and 25 tubes were eluted out with 1X PBS (pH 7.2) with specific intervals and analyzed further for absorbance.

### Spectrophotometry analysis

**Figure 1:**
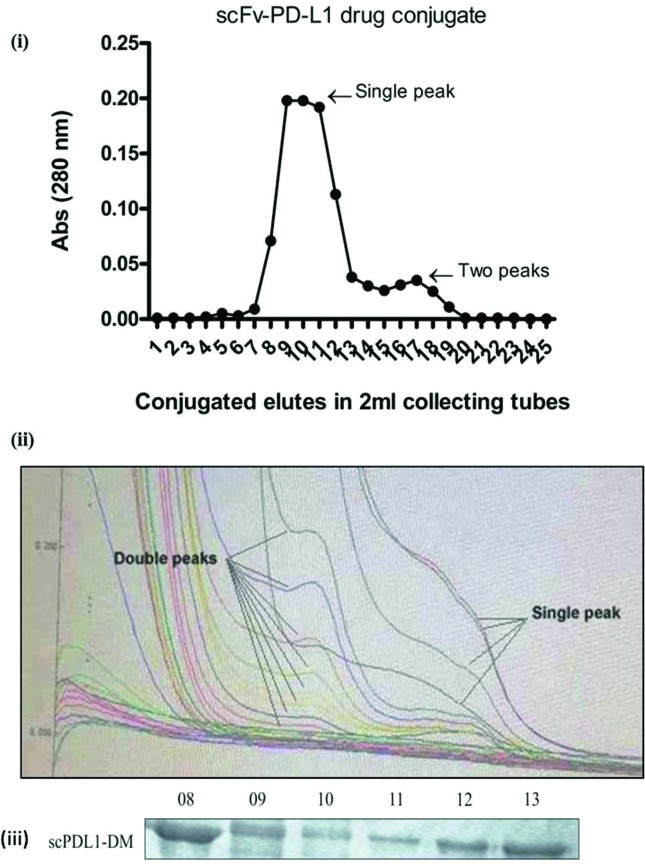
Spectrophotometry chromatogram peaks absorbance. (i): Absorbance wavelength of scFv-PD-L1 drug conjugates after the Sephadex G-25 column at 280 nm was calculated. A total of 25 tubes were collected after loading and elution with 1X PBS (pH 7.2). The maximum wavelength was found from 8 to 11 with a single peak location. From tubes 12 to 18 the peaks were detected in two different positions. (ii): Average drug to antibody ratios determination from peaks location that indicates a single observed location at four different points, may predict drug conjugations with scFv-PD-L1. The double peaks indicate unconjugated drugs. (iii): SDS-PAGE analysis showing results of eluted scPDL1-DM1 drug conjugate after G250 column purification.

Homogenous purified drug conjugate purity was calculated from absorbance and peaks determination at 280 nm as shown in Figure 1(i) & (ii). In the chromatograms, the two major peaks were observed corresponding to the scFv light chain and heavy chain that were clearly right shifted. These species were considered as unconjugated chains and the DAR values may be underestimated due to impurities or hydrophobicity. The other single peak was observed with high absorbance and was considered as conjugated drugs loaded with 0-8 drug ratios. Absorbance was calculated as shown in Table 1. Further elucidation needed to quantify the DAR, binding sites and kinetic studies of the drug. SDS-PAGE analyses were also performed initially for eluted scPDL1-DM1 after the G-25 Sephadex column as shown in Figure 1 (iii).

**Table 1:** Absorbance of the eluted drug after Sephadex G-25 column size exclusion purification

Abs	1	2	3	4	5	6	7	8	9	10	11	12
**280**	0.013	0	0	0.009	0.005	0.003	0.009	0.071	0.198	0.192	0.113	0.048
**252**	0.015	0	0	0.012	0.007	0.004	0.01	0.108	0.55	0.588	0.406	0.232
**13**	**14**	**15**	**16**	**17**	**18**	**19**	**20**	**21**	**22**	**23**	**24**	
**280**	0.038	0.03	0.026	0.031	0.035	0.025	0.011	0.003	0.001	0	0	0
**252**	0.177	0.116	0.079	0.058	0.045	0..031	0.022	0.013	0.007	0	0	0.003
**25**	**26**	**27**	**28**	**29**	**30**	**31**	**32**	**33**	**34**	**35**	**36**	
**280**	0.001	0.001	0.001	0.001	0.006	0	0	0	0	0	0.001	0
**252**	0.005	0.001	0.002	0.002	0.009	0	0	0	0	0	0.001	0

### SDS-PAGE analysis

**Figure 2:**
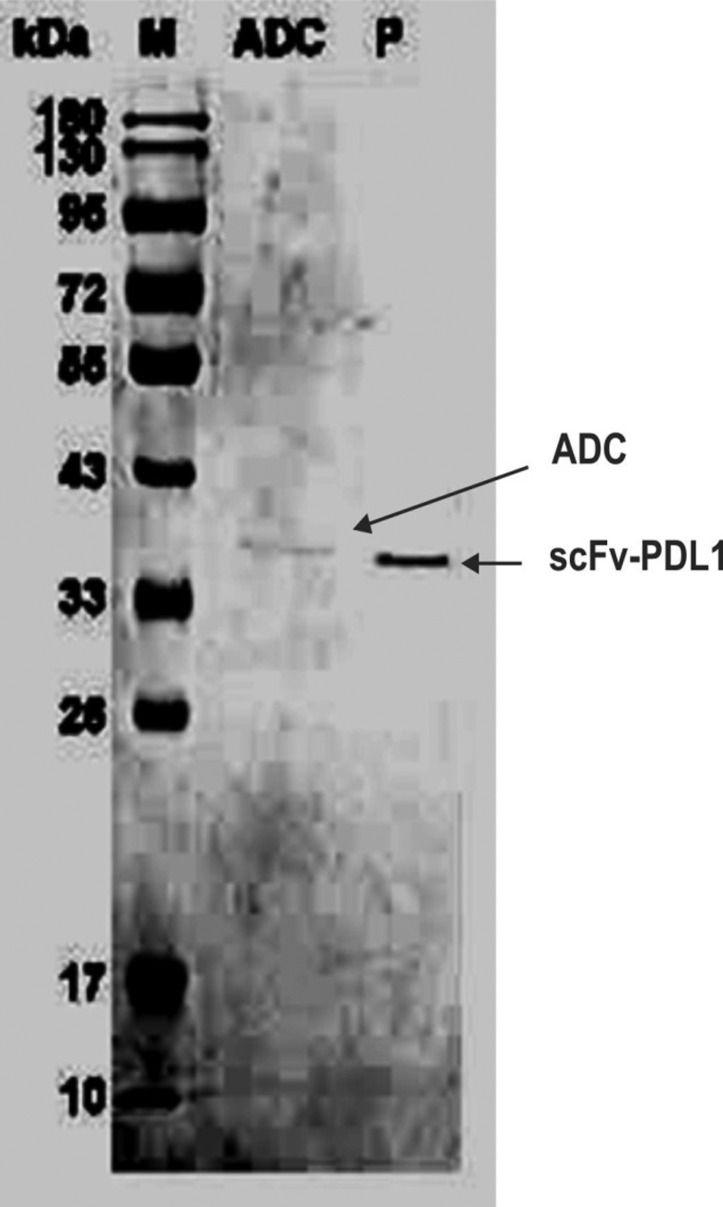
SDS-PAGE determination of conjugated drug. The figure shows a slight increase in 1kDa in molecular weight compared to proteins. M indicates the molecular marker, ADC shows the conjugated molecules and P shows purified scFv-PD-L1 expressed proteins. 12% gel was used for differentiation.

Protein samples and scPDL1-DM1 were analyzed by sodium dodecyl sulfate-polyacrylamide gel electrophoresis (SDS-PAGE) on 12% gel. The gel was stained with Coomassie brilliant blue dye and mean densities of proteins and drug conjugates were determined. SMCC-DM1 possesses a molecular weight of 1 kDa to predict a slight increase of localization of protein bands on the gel. The slight increase in band size showed a slight change for the conjugated proteins while the unconjugated proteins have no change with a molecular weight of approximately 34 kDa as shown in Figure 2.

### Western blotting

**Figure 3:**
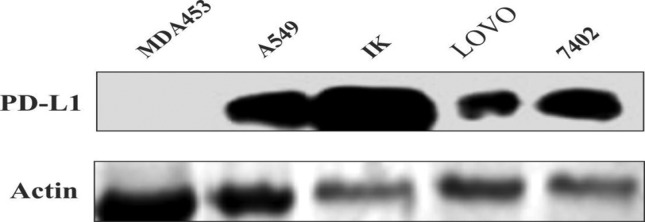
Western blot analysis of PD-L1 positive cancer cell lines. Total proteins were isolated from cancer cell lines and analyzed for PD-L1 signals. Proteins were isolated with RIPA buffer and extracted on 12% SDS-PAGE for differentiation and transferred to a membrane. The four cancer cell lines show positive anti-PD-L1 signals while MDA453 was found negative.

BEL-7402 (Hepatic Carcinoma), A549 (Adenocarcinoma, Lung Cancer), IK (Ishikawa, Endometrium cancer), LOVO (Colorectal cancer) and MDA453 cancer cells were cultured in 6 wells plate and total proteins were extracted by using RIPA buffer. Western blot analysis revealed the differences between PD-L1 positive and negative cell lines. It was concluded that all cells, except MDA453, showed positive signals for PD-L1 as shown in Figure 3. This preliminary study aids *in vitro* analysis and immunofluorescence determination for further investigations.

### In vitro studies

**Figure 4:**
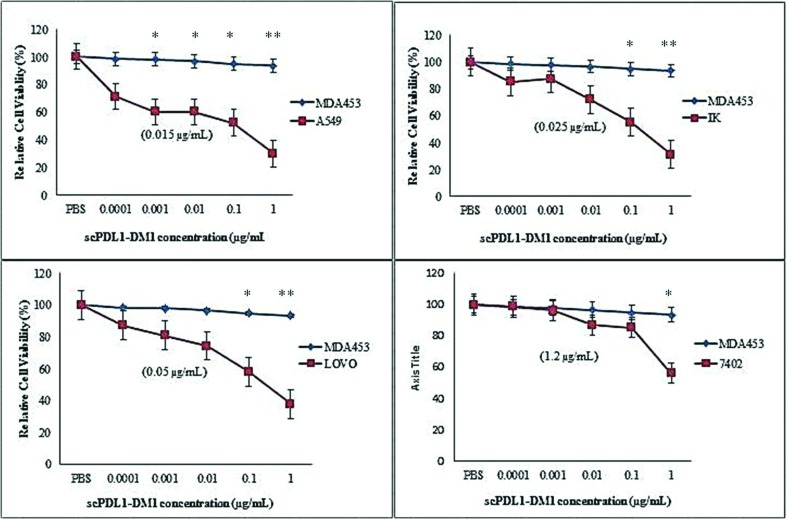
Cell viability detection after 48 hours by MTT analysis. ScPDL1-DM1 conjugate induces a potent antiproliferative effect in PD-L1 positive tumor cells (A549, IK, LOVO, and 7402) but has no effect on PD-L1 negative MDA453 cells. MDA453 cells were used as control. These cells were incubated in 96 wells plate in drug conjugate for 48 hours. PBS was also used as a negative control. The IC50 values for each cell line are shown in parentheses. The test experiments were repeated three times as indicated in standard bars. Oneway ANOVA was used to analyze the cell viability after 48 hours of cell treatment, **p<0.01, *p<0.05.

**Figure 5:**
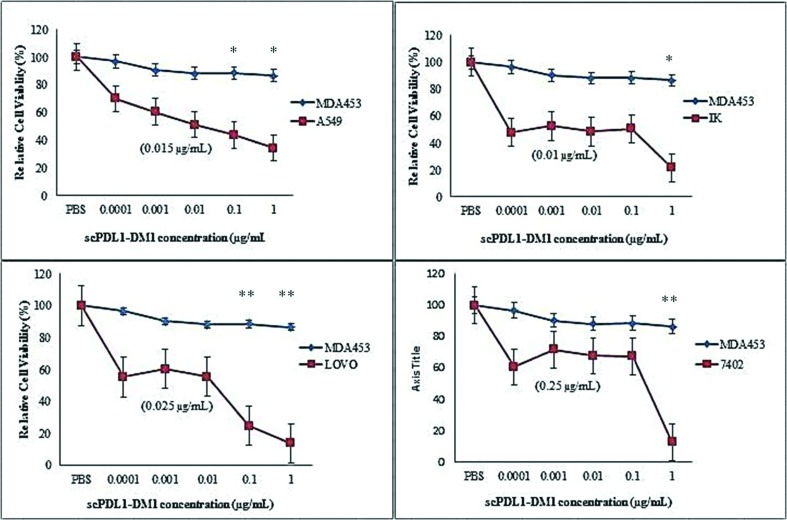
Cell viability detection after 60 hours by MTT analysis. ScPDL1-DM1 conjugate induces cell lysis and apoptosis effect on A549, IK, LOVO, and 7402 tumor cell lines but has no effect on PD-L1 negative MDA453 cells. MDA453 cells were used as control. These cells were incubated in 96 wells plate in drug conjugate for 60 hours. PBS was also used as a negative control. The IC50 values for each cell line are shown in parentheses. The test experiments were repeated three times as indicated in standard bars. One-way ANOVA was used to analyze the cell viability after 48 hours of cell treatment, **p<0.01, *p<0.05.

By employing conjugation strategies we were fully able to administer the cytotoxic activity of scPDL1-DM1 conjugates on cancer cells. In vitro activity analysis was conducted on BEL-7402 (Hepatic Carcinoma), A549 (Adenocarcinoma, Lung Cancer), IK (Ishikawa, Endometrium cancer), LOVO (Colorectal cancer) and MDA453 cell lines using 96 wells plate at different time frame (48, 60 and 72 hours). The cell concentrations were kept constant up to 5000 cells per well in media supplemented with different concentrations of drug conjugates (0.0001, 0.001, 0.01, 0.1 and 1 μg/mL). The metabolic activity harassment of cancer cells was calculated by measuring the absorbance at 570 nm and the final cytotoxicity was calculated with MTT analysis. The cytotoxic activity of drug conjugate was calculated after 48, 60 and 72 hours as shown in figures 4, 5, & 6. The enhanced potency of drug conjugates was observed at 72 hours induction with 1 μg/mL concentrations of the drug against all four PD-L1 positive cells but no such positive activity was detected against PD-L1 negative MDA453 cell lines. The ic50 values were determined in separate experiments as shown in parentheses. The cells were treated at different concentrations of drug and absorbance was determined. Almost all tested cells showed sensitivity except MDA453. The maximum activity was observed against LOVO colorectal cancer cell lines. The test experiments were repeated three to five times and data were plotted by using One way ANOVA analysis of variance with GraphPad Prism 5 (GraphPad Software; Inc: La Jolla, CA) software to evaluate the cytotoxic activity of the drug.

**Figure 6:**
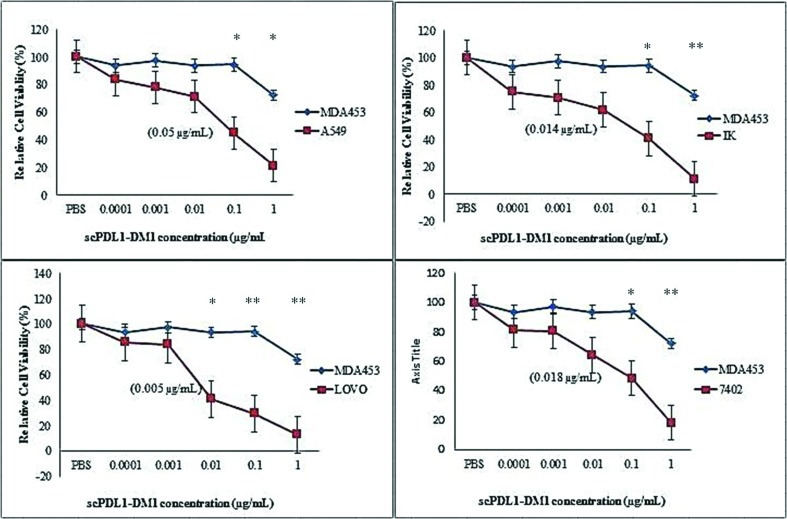
Cell viability detection after 72 hours by MTT analysis. After incubation of 72 hours, the scPDL1-DM1 conjugate shows the potent anti-proliferative effect on PD-L1 positive tumor cells (A549, IK, LOVO, and 7402) but has no effect on PD-L1 negative MDA453 cells. MDA453 cells were used as control. These cells were incubated in 96 wells plate in drug conjugate for 72 hours. The IC50 values for each cell line are shown in parentheses. The test experiments were repeated three times as indicated in standard bars. One-way ANOVA was used to analyze the cell viability after 48 hours of cell treatment, **p<0.01, *p<0.05. Maximum activity was observed after 72hrs of incubation.

### Surface binding specificity and trafficking analysis

**Figure 7:**
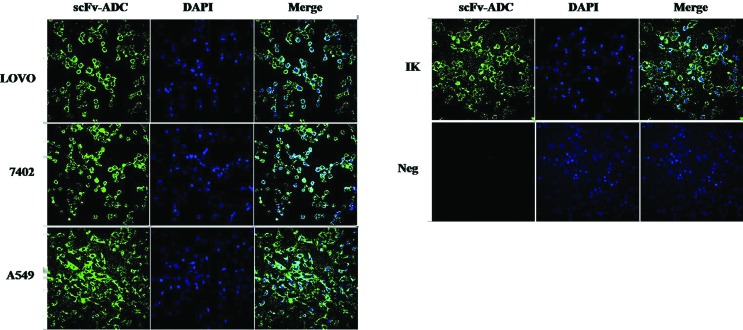
Immunofluorescence analysis of the conjugated scFv-PD-L1 drug. Cells were seeded onto glass coverslips and washed gently with 1X PBS (pH 7.2) and fixed with 4% paraformaldehyde for 20 minutes. Drug conjugate was applied and incubated for one night at room 4°C. After washing coverslips were loaded with FITC labeled rabbit anti-human IgG (H+L) antibody (dilution 1:1000) for 1 hour at 37°C. DAPI (dilution 1:1000) was added to stain the nucleus and were visualized under a 63x oil immersion objective using a Zeiss fluorescence microscope. Positive cells showed fluorescence while no signals were found on the negative cell surface with scFv-PD-L1 drug conjugates.

The binding specificity of positive cells was obtained by investigating the drug-conjugate binding ability to these cell surfaces. We first confirmed the PD-L1 transcript expression in selected cancer cell lines by using western blot analysis and immunofluorescence analysis. MDA453 cancer cell line was used as a control and was always shown to be negative as shown in Figure 7. The flow cytometry analysis was conducted to evaluate the surface binding and trafficking studies. Cells were loaded on cover slides and fixed with 4% paraformaldehyde. The cells were fixed and washed. Cells were loaded with scPDL1-DM1, followed by rabbit anti-human IgG (H+ L) FITC conjugated antibodies. The concluded results indicate the positive fluorescence for drug conjugates. The negative MDA453 was deprived of such visible signals. The PD-L1 positive control antibody was also applied to verify the positive conjugations as shown in Figure 7. These results suggested that the drug can be utilized as a potent inhibitory tool for cancer therapy by targeting the PD-L1 surface antigens with further evaluations.

**Figure 8:**
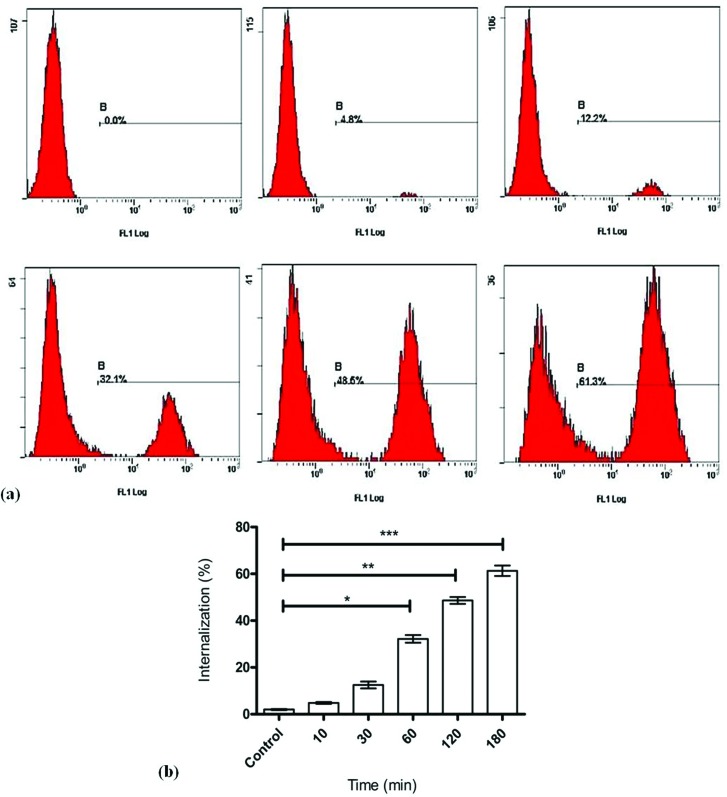
Intracellular trafficking of the conjugated drug through Flow-Cytometry analysis. (a): Six different panels show the intracellular trafficking mechanism of the scFv-PD-L1 drug against A549 hepatocellular carcinoma as a model. The flow rate was conducted through Flow Cytometry analysis. The upper panel from left to right indicates 0 minutes, 10 minutes and 30 minutes of incubation. The lower panel from left to right shows 1 hour, 2 hours and 3 hours of incubation time. The percent increase was calculated that shows elevated trafficking mechanisms after 2 hours and reaches a maximum at 3 hours of incubation. (b): After each time point, the proportion of internalized drug conjugates was measured. Data were generated from three independent experiments. One-way ANOVA was used to analyze the maximum trafficking studies, ***p<0.001, **p<0.01, *p<0.05.

**Figure 9:**
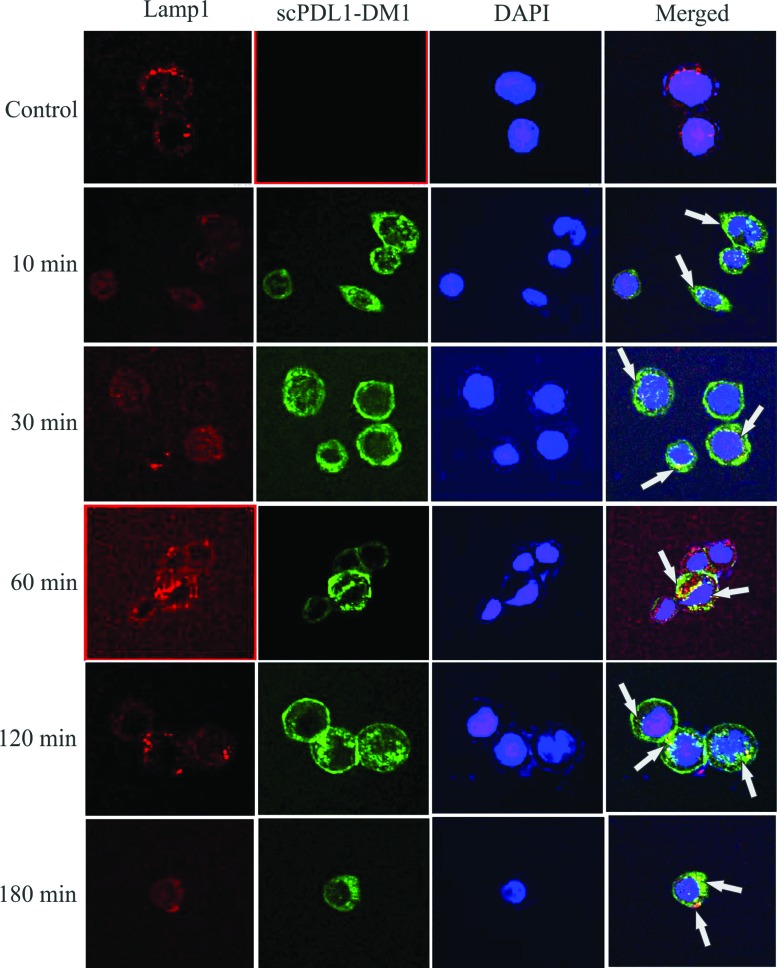
Immunofluorescence analysis of scFv-PD-L1 drug trafficking. A549 cells were loaded on cover slides and incubated with the drug at different time intervals for surface localization and trafficking studies followed by FITC conjugated second antibody. Mouse monoclonal Lamp1 antibody was loaded to bind with lysosomes and counterstained with anti-mouse secondary antibody Alexa 488 conjugated. DAPI was loaded to stain the nuclei. Compparative accumulation of fluorescent signals within cellsindicates scPDL1-DM1 drug conjugates. In the first panel, there was no overlay in the merge with no signals on surfaces of control cell line. At 10 minutes interval, the binding start and trafficking were observed. After 2 hours maximum trafficking to lysosomes was observed with slight returning back of drug to surface. At 3 hours of incubation the surface localization increase that indicates the maximum returning back of drugs to the cell surface. The arrows indicate overlay of scPDL1-DM1 and trafficking in lysosomes. Photographs were taken with 63x oil immersion objective using Zeiss fluorescence microscope (Zeiss, Germany) in the core facility of Zhejiang University China.

The flow cytometry and immunofluorescence analysis showed maximum localization and intracellular trafficking after 2 hours of incubation with more than 50% intensity by using A549 cancer cell lines (Figure 8). After 3 hours of incubation, the trafficking of drug conjugates was highly significant and recorded 61.3% intensity. At zero intervals the trafficking was found 0% also fluorescence did not show any visible overlay formation in lysosomal trafficking. The internalization mechanism was done very soon after 10 minutes and reached the elevated level after 30, 60, 120, and 180 minutes in A549 cancer cell line as shown in Figure 8(b). The fluorescence signals also predict the visible appearance of drug binding on surfaces after 10 minutes with maximum trafficking after two hours of incubation as shown in Figure 9. After each time point, the proportion of antibody remaining in the cell surface was visualized with flourochrome labeled antibody. These results confirmed that internalization mostly relays on antigen receptor-mediated endocytosis. All these analyses confirmed the binding affinity, bioactivity confirmations and trafficking studies of scPDL1-DM1 drug conjugates that may be considered for further evaluations in therapeutic studies.

## Discussion

A novel strategy was constructed to design drug conjugate consuming scFv fragments against the PD-L1 surface antigen. High-affinity chain fragments were chosen and amplified further for drug development ought to better efficiency in cancer treatment preliminary experiments. The screening platform allowed the sympathy of antibody candidates for drug conjugate in the easy manipulated single step conjugation methodology. The scPDL1-DM1 was successfully proliferated and identified for active cytotoxicity analysis against PD-L1 positive cells. scFv fragments were abundantly utilized for full-length antibody development but never reported for direct conjugation strategies. The purity of monoclonal antibody was similar to various FDA approved antibodies which can be utilized further for the therapeutically applicable approach (Kaja *et al.*, 2011; Chen *et al.*, 2011; Amedee, 2011). Most classical ADC has been designed by binding cytotoxic drugs with lysine or cysteine residues with a full-length antibody that results in an unpredictable heterogeneous mixture of limited pharmacokinetics applications. The relatively high molecular weight of approximately 150 kDa makes the ADC difficult to penetrate in solid tumors and persistent hold in non-target tissues (Junutula *et al.*, 2008, Wang *et al.*, 2005, Hamblett *et al.*, 2004). As it was observed that the maximum time acquired by the newly generated scPDL1-DM1 for endocytosis was 2 hours incubations. So, efficient endocytosis and intracellular trafficking will occur avoiding the immune response. Additionally, it was reported that α-kappa-ETA conjugate persists suitable internalizing tools derived from phage display libraries. The isolated Fab fragment was converted into the full antibody by the reduction of the human light antibody fragment with other high specific domains (Hust *et al.*, 2007; Thie *et al.*, 2008).

Heterogeneity of loaded drugs challenges the therapeutics application and in vivo associated effects. It was found that lysine residues share approximately 40 different binding sites that result in a potentially abundant amount of antibody-drug conjugates (Wang *et al.*, 2005). Cysteine residue reduces partially the disulfide bonds utilized in more than 100 ADC molecules with 0-8 DAR value. The inter-chain disulfide linkage results in quaternary antibody structure modification that becomes a challenge in therapeutic application. Our design studies will prevent heterogeneity issues by direct attachment with targeted sites that also retain all sites.

The PD-L1 conjugated molecule utilizes scFv that are quite tiny to penetrate in tumors cell with elevated sharp cytotoxic effects. These ADCs can be obtained easy, quickly and efficiently that provides an inexpensive approach for the optimization of drug conjugates (Hussain *et al.*, 2011; Woitok *et al.*, 2016; Hussain *et al.*, 2013). We have tested the binding competency of scPDL1-DM1 with PD-L1 positive tumor cells. Several other site-directed conjugated methodologies are being reported for full-length antibody production with prominent clearance applications (Kline *et al.*, 2015; Polakis, 2016). Our predicted results showed more than 60% cytotoxicity against positive cells. The generation of scPDL1-DM1 was initially confirmed by SDS-PAGE, spectrophotometry and fluorescence microscopy. SDS-PAGE indicates a slight increase in band location size as compared to unconjugated protein. The conjugation proliferation was established that indicated two different peeks absorbance. Single peak showed the conjugated drugs while the other peak predicted the unconjugated entity. The unconjugated absorbance was localized at two different positions that discriminate the unconjugated drug from conjugated one. The four different peak locations as shown in Figure 1, may predict the drug-antibody ratios of 0, 1, 2, 4 and 8 but further confirmation needed to evaluate the DAR and its specific binding sites. As mechanistic studies of ADC endocytosis and internalization was reported that precise attachment of drug occurs with its target antigens, and showed an easy approach to lysosome through trafficking mechanism (Dal Corso *et al.*, 2018). We incubated the targeted cells at different intervals of times with scPDL1-DM1 and found that at 2 hours of incubation almost all conjugated molecules have got access to cells that may be utilized as a potent incubation time frame for *in vivo* studies. The generated drug can be utilized as a potent tool for site-specific conjugation, predicting specificity *in vitro* activities with extended range against PD-L1 positive cancer cells and can be utilized for further *in vivo* testing and clinical therapeutics development. Our studies consist of few limitations including DAR, binding sites identification of drug, half-life determination and *in vivo* study analysis. The strategies need development and efficiency improvement to enhance direct anti-tumor activity and bio-therapeutics enrichment in cancer therapy.
